# Discovery of prognostic biomarkers for predicting lung cancer metastasis using microarray and survival data

**DOI:** 10.1186/s12859-015-0463-x

**Published:** 2015-02-21

**Authors:** Hui-Ling Huang, Yu-Chung Wu, Li-Jen Su, Yun-Ju Huang, Phasit Charoenkwan, Wen-Liang Chen, Hua-Chin Lee, William Cheng-Chung Chu, Shinn-Ying Ho

**Affiliations:** 1 Institute of Bioinformatics and Systems Biology, National Chiao Tung University, Hsinchu, Taiwan; 2 Department of Biological Science and Technology, National Chiao Tung University, Hsinchu, Taiwan; 3 Division of Thoracic Surgery, Department of Surgery, Taipei Veterans General Hospital, Taipei, Taiwan; 4 Institute of Systems Biology and Bioinformatics, National Central University, Taoyuan, Taiwan; 5 Institute of Molecular Medicine and Bioengineering, National Chiao Tung University, Hsinchu, Taiwan; 6 Department of Computer Science, Tunghai University, Taichung, Taiwan

**Keywords:** Distant metastasis, Genetic algorithm, Lung cancer, Microarray, Prognostic biomarker, Survival curve

## Abstract

**Background:**

Few studies have investigated prognostic biomarkers of distant metastases of lung cancer. One of the central difficulties in identifying biomarkers from microarray data is the availability of only a small number of samples, which results overtraining. Recently obtained evidence reveals that epithelial–mesenchymal transition (EMT) of tumor cells causes metastasis, which is detrimental to patients’ survival.

**Results:**

This work proposes a novel optimization approach to discovering EMT-related prognostic biomarkers to predict the distant metastasis of lung cancer using both microarray and survival data. This weighted objective function maximizes both the accuracy of prediction of distant metastasis and the area between the disease-free survival curves of the non-distant and distant metastases. Seventy-eight patients with lung cancer and a follow-up time of 120 months are used to identify a set of gene markers and an independent cohort of 26 patients is used to evaluate the identified biomarkers. The medical records of the 78 patients show a significant difference between the disease-free survival times of the 37 non-distant- and the 41 distant-metastasis patients. The experimental results thus obtained are as follows. 1) The use of disease-free survival curves can compensate for the shortcoming of insufficient samples and greatly increase the test accuracy by 11.10%; and 2) the support vector machine with a set of 17 transcripts, such as CCL16 and CDKN2AIP, can yield a leave-one-out cross-validation accuracy of 93.59%, a test accuracy of 76.92%, a large disease-free survival area of 74.81%, and a mean survival prediction error of 3.99 months. The identified putative biomarkers are examined using related studies and signaling pathways to reveal the potential effectiveness of the biomarkers in prospective confirmatory studies.

**Conclusions:**

The proposed new optimization approach to identifying prognostic biomarkers by combining multiple sources of data (microarray and survival) can facilitate the accurate selection of biomarkers that are most relevant to the disease while solving the problem of insufficient samples.

**Electronic supplementary material:**

The online version of this article (doi:10.1186/s12859-015-0463-x) contains supplementary material, which is available to authorized users.

## Background

Primary lung cancer is very heterogeneous in its clinical presentation, histopathology, and treatment response [1]. Differentiating between an occurrence of a new primary lung cancer and a recurrence of lung cancer is often difficult. Conventionally, lung cancers have been divided into non-small-cell lung cancer (NSCLC) and small-cell lung cancer (SCLC). The stage of each cancer is the most significant predictor of survival. Cancer metastasis and the emergence of drug resistance are the major causes of the failure of treatment for lung cancer. Thus, therapy for lung cancer that takes into account distant metastasis and drug resistance is an emerging field of research. Prognostic biomarkers are expected to be useful in predicting the probable course of lung cancer metastases, and they importantly affect the aggressiveness of therapy. Some new promising strategies for biomarker discovery include microarray-based profiling at the DNA and mRNA levels, and mass-spectrometry-based profiling at the protein and peptide levels [2]. The combination of multiple biomarkers is generally agreed to increase diagnostic sensitivity and specificity over the use of individual markers.

During cancer progression, some tumor cells acquire new characteristics, such as over-expression of epithelial-mesenchymal transition (EMT) markers, and undergo profound morphogenetic changes. EMT is a process in which epithelial cells lose their cell polarity and cell-cell adhesion, and gain migratory and invasive properties, becoming mesenchymal cells [3,4]. EMT plays an important role in cancer progression and provides a new basis for understanding the progression of carcinoma towards dedifferentiated and more malignant states [3,4]. Additionally, EMT affects cancer cell invasion, resistance to apoptosis, and stem cell features [5]. Growth factors [6,7], ligand-dependent nuclear receptors [8], transcription regulators [3,9], cytokine [7,10], and kinase [11,12], which are potential regulators that are related to EMT have been identified in the literature. Signaling pathways that are activated by intrinsic or extrinsic stimulation converge on the transcriptional factors and regulate phenotypic changes of cancer cells [9].

DNA microarrays perform the simultaneous interrogation of thousands of genes and provide an opportunity to measure a tumor from multiple perspectives. Microarray-based techniques generally provide detailed observations at gene activities in tumors and generate opportunities for finding therapeutic targets. As a high-throughput technology at the molecular level, DNA microarray-based methods have clear advantages over traditional histological examinations and have been extensively used in cancer research to predict more accurately clinical outcomes and potentially improve patient management. Studies indicate that microarray techniques greatly facilitate accurate tumor classification and predicted outcome in terms of, for example, tumor stage, metastatic status, and patient survival, offering some hope for personalized medicine [13-15].

Methods for predicting lung cancer metastasis involve feature (gene) selection and classifier design. Feature selection identifies a subset of differentially-expressed genes that are potentially relevant to distinguishing different classes of samples [16]. One of the central difficulties in investigating microarray classification and gene selection is the availability of only a small number of samples, compared to the large number of genes in a sample [16]. Hierarchical clustering [17] is one of the most commonly used approaches in microarray studies. However, hierarchical clustering (or any purely correlative technique) cannot alone provide a rational biological basis for disease classification [18]. Generally, univariate analysis is conducted to reduce feature size and, then, a support vector machine (SVM) [19] or maximum likelihood classification [20] with an effective feature selection method is used to identify a small set of informative genes. The most challenging task is to avoid overfitting a small number of samples, resulting in the poor performance of independent tests.

In this work, the medical records of 78 patients with lung cancer and a follow-up time of 120 months reveal significant difference between the disease-free survival times of the 37 non-distant- and the 41 distant-metastasis patients. To solve the problem of insufficient samples, this work proposes a novel optimization approach to discovering EMT-related prognostic biomarkers for predicting the distant metastasis of lung cancer using both microarray and survival data. The proposed optimal gene selection method incorporates gene expression profiles and their corresponding disease-free survival curves of patients to design a fitness function for using an intelligent genetic algorithm [21]. The set of 78 samples is used to identify a set of gene markers and an independent cohort of 26 samples is used to evaluate the identified biomarkers. The experimental results show that disease-free survival curves can compensate for the insufficient samples and the SVM with a set of 17 transcripts can yield high prediction accuracies of distant metastasis and disease-free survival time. The putative biomarkers for predicting the distant metastasis of lung cancer are examined using relevant signaling pathways to reveal the potential of biomarkers.

## Methods

### Data sets

#### RNA isolation and microarray platform

Illumina Sentrix-6 Whole-Genome Expression BeadChips are relatively new microarray platforms, that have been used in many microarray studies in the past few years [22]. Physically, each Sentrix-6 BeadChip consists of 12 equally-spaced strips of beads. Each pair of adjacent strips comprises a single microarray and is hybridized with a single RNA sample [22]. The used microarray is Illumina of HumanWG-6 BeadChip Kit Support. Fresh-frozen specimens were removed from liquid nitrogen and homogenized using TissuLyzer in RLT buffer of RNeasy isolation kit, both from Qiagen. Total RNA was extracted from fresh-frozen tumors followed the manufacturer’s suggestion, purified by RNeasy mini kit, and checked by NanoDrop spectrophotometer and Agilent Bioanalyzer for quantity and quality. Biotin labeled cRNA was prepared from Illumina TotalPrepTM RNA amplification kit, Life Technologies. One and half ug cRNA was hybridized to the Illumina Multi-sample Human WG-6 v3.0 chip according to manufacturer’s instructions. Globe normalization was used to normalize for signal intensity of chips. This microarray of a sample has 48,803 transcripts.

#### Cohorts of lung cancer

The dataset of 78 lung cancer samples, comprising 37 non-distant- and 41 distant-metastasis samples as well as their corresponding disease-free survival time, comes from Taipei Veterans General Hospital (TVGH) in Taiwan. This work developed prognostic models using these 78 samples. To evaluate the potential of putative biomarkers and the gene-discovery method for identifying a small set of genes that can be used to predict distant metastasis of lung cancer, a cohort of 26 samples, comprising 6 non-distant- and 20 distant-metastasis patients of TVGH, is utilized as an independent test dataset. This study as well as the tissue procurement protocol were approved by the Institutional Review Board of TVGH (VGHIRB No. 2013-04-015 AC), and written informed consent was obtained from all patients. The datasets of 78 and 26 samples consisting of microarray data and disease-free survival time that the patient identifiers have been removed are given in Additional file 1: Table S1 and Additional file 2: Table S2, respectively.

#### Characteristics of cohorts

**Figure 1:**
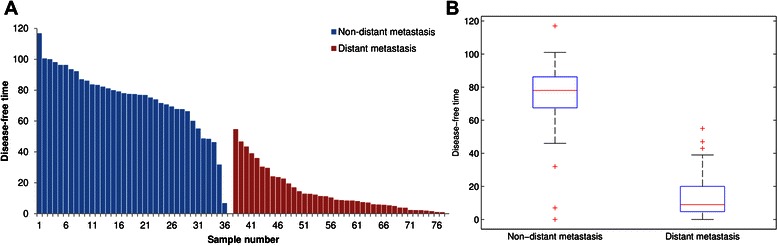
**Disease-free survival times for 78 lung cancer patients with a follow-up time of 120 months. (A)** The mean times of disease-free survival for non-distant and distant metastasis are 73.08 and 14.02 months, respectively. **(B)** The box plots of non-distant and distant metastasis. The p-value of *t*-test is 7.99E-22 (p < 0.001) suggesting that distant metastasis is highly correlated to patients’ survival.

Patient survival is a major clinical parameter that is used to evaluate the efficacy of a particular therapy. Disease-free survival, used herein, is defined as the time between surgery and the occurrence of an event (death or distant metastasis). The censored data are that when the event did not occur, and the survival time is that between surgery and the last follow-up date. Figure 1 shows statistics concerning disease-free survival times for 78 lung cancer patients with a follow-up time of 120 months. The mean times of disease-free survival for non-distant and distant metastasis are 73.08 and 14.02 months, respectively. A *t*-test with p-value = 7.99E-22 reveals a significant difference (p < 0.001) between the disease-free survival times of the 37 non-distant- and the 41 distant-metastasis patients. The result suggests that distant metastasis is strongly correlated to patients’ survival. Some characteristics of these 78 patients were summarized in Table 1. From the results of Fisher’s exact test, there was no significant association between distant metastasis and the interested factors such as age, sex, smoking, tumor size, pN and pM status, histologic type, and differentiation (p > 0.05). Notably, there was a strong association between distant metastasis and pathologic stage (p = 3.0E-5).

**Table 1:** **Selected characteristics of participants according to NSCLC**

**Characteristics**		**Number (N = 78)**	**%**	**Metastasis**		***p*** ^**a**^
				**+**	**-**	
**Age**						
	≧65	49	63	25	24	0.64
	<65	29	37	17	12	
**Sex**						
	Male	56	72	28	28	0.32
	Female	22	28	14	8	
**Smoking index**						
	≧20 pack-year	35	45	17	18	0.49
	<20 pack-year	43	55	25	18	
**Histologic type**						
	Squamous cell	25	32	11	14	0.46^b^
	Adenocarcinoma	46	59	25	21	
	Large cell	5	6	4	1	
	Other cell type	2	3	2	0	
**Differentiation**						
	Well	5	6	1	4	
	Moderate	51	65	28	23	0.80^b^
	Poor	22	28	13	9	
**Tumor size**						
	≧5 cm	18	23	11	7	0.59
	<5 cm	59	76	31	28	
**pN status**						
	Positive	54	69	25	29	0.09
	Negative	25	32	17	7	
**pM status**						
	Positive	5	6	5	0	0.20
	Negative	73	94	37	36	
**Pathologic stage**						
	Stage I	36	46	10	26	3.0E-5^c^
	Stage II	11	14	7	4	
	Stage III	26	33	20	6	
	Stage IV	5	6	5	0	

^a^The two-sided *p-*values were calculated by Fisher's exact test.

^b^The *p*-values were calculated using the two variables with the largest numbers of patients.

^c^The *p*-value was calculated to measure the association between two variables (Stage I and Stages II, III and IV).

#### EMT-related transcripts

Ingenuity Knowledge Base (IKB; http://www.ingenuity.com) is a repository of biological interactions and functional annotations that are created from millions of individually modeled relationships among proteins, genes, complexes, cells, tissues, metabolites, drugs, and diseases. IKB provides comprehensive, species-specific knowledge about the function and regulation of genes, tissue and cell line expression patterns, clinical biomarkers, subcellular locations, mutations, and disease associations. First, EMT-related genes annotated as “growth factor”, “ligand-dependent nuclear receptor”, “transcription regulator”, “cytokine”, or “kinase” in the IKB are identified. As a result, 4,314 transcripts are identified in the HumanWG-6 microarray data, comprising 233 transcripts of growth factor, 103 transcripts of ligand-dependent nuclear receptor, 2,465 transcripts of transcription regulator, 276 transcripts of cytokine, and 1,237 transcripts of kinase. Second, univariate *t*-test analysis is used to calculate the *p*-values of individual transcripts between two classes. The genes with a very small *p*-value are useful in predicting distant metastasis. There are 474 top-ranked transcripts according to the p-value selected from the 4,314 EMT-related transcripts. The training dataset of 78 samples with the 474 EMT-related transcripts is used to identify a small set of prognostic biomarkers that is predictive of the distant metastasis of lung cancer.

### Methods

#### Disease-free survival area

The Kaplan-Meier survival curves reveal a significant difference between the survival times of patients in the two classes. The disease-free survival area is calculated between the two survival curves from zero to the maximum disease-free survival time (117 months in this work). The disease-free survival area is represented as a percentage, which is the ratio of this area to the maximum area. The gene set with a large disease-free survival area is expected to have a strong ability to distinguish a non-distant metastasis from a distant metastasis of lung cancer.

#### Fitness function

Microarray data contain valuable information about a huge gene set but often suffer from a small number of available samples. In the design of classifiers with gene selection, there exist numerous candidate sets of genes that can achieve high training accuracies for predicting distant metastasis. However, most of the candidate gene sets have relatively low accuracies of independent tests that result in overtraining. The right sets of biomarkers should have high prediction accuracies for both training and test datasets. To cope with the overtraining problem, it is essential to identify the right sets of biomarkers by discarding the gene sets of overtraining. Recently obtained evidence reveals that distant metastasis from lung cancer is detrimental to patients’ survival [23]. Moreover, Figure 1 suggests that distant metastasis is strongly related to patients’ survival. Thus, it is hypothesized that the right set of biomarkers can also be used to effectively predict patients’ survival. This work proposes a hybrid approach that uses two kinds of resources, microarray data and the disease-free survival data, to identify a set of biomarkers.

The fitness function provides the only means by which genetic algorithms (GAs) optimize all system parameters that are encoded in a GA-chromosome. The three objectives of designing the predictor of lung cancer metastasis and discovering a set of genes using GA-based optimization methods are as follows. The first objective is to maximize the classification accuracy (denoted as *Acc*) of the SVM classifier; the second is to maximize the disease-free survival area (denoted as *Asurv*), and the last is to identify a small set of informative genes. The values of *Acc* and *Asurv* are in the range [0, 1]. The two maximum objectives without conflicting each other can be combined into a weighted objective function *f*(*G*) as follows.

1 $$ \mathrm{Max}f(G)=w\times Acc + \left(1\hbox{--} w\right)\times Asurv $$

where *w* denotes a positive weight in the range [0, 1] which is determined according to the preferences for individual objectives, and *G* denotes the selected gene set. Generally, maximizing *f*(*G*) is the major objective and the number of selected genes is restricted within a relatively small range (as will be discussed in the next section). If *w* = 1.0, then the fitness function degenerates to the conventional one that uses no clinical outcome (disease-free survival curve). The fitness function using the overall accuracy *Acc* is suitable for balanced datasets (almost equal populations of metastatic positive and negative samples). When applying to imbalanced datasets, random over-sampling methods such as SMOTE which is short for Synthetic Minority Over-sampling Technique [24] can be used to adjust these datasets to balanced datasets.

#### Gene selection using IBCGA

Selecting a minimal number of informative genes while maximizing the prediction performance of distant metastasis is a bi-objective 0/1 combinatorial optimization problem. This work propose a novel method for identifying a small number *m* of informative genes from a large number *n* of candidate genes for prediction and biomarker discovery based on an inheritable bi-objective combinatorial genetic algorithm (IBCGA) [25] with SVM classifiers. IBCGA has been previously used to identify a small set of properties from 531 physicochemical properties for predicting the immunogenicity of MHC class I binding peptides [26]. Feature selection is a combinatorial optimization problem C(*n*, *m*) with a huge search space of size C(*n*, *m*) = *n*!/(*m*!(*n*-*m*)!)). The IBCGA uses an intelligent genetic algorithm (IGA) [21] with an inheritance mechanism [25] to search efficiently for the solutions *S*_r_ to C(*n*, *r*) and *S*_r+1_ to C(*n*, *r* + 1) by inheriting the good solution *S*_r_. IGA that is based on orthogonal experimental design uses a divide-and-conquer strategy and a systematic reasoning method rather than a conventional generate-and-go method to solve efficiently the large-scale combinatorial optimization problem. The SVM-based training model uses the prediction performance of leave-one-out cross-validation (LOOCV) as the fitness function in using IBCGA with the whole training set.

The input for the SVM-based model design procedure is a training dataset that is composed of two classes (distant and non-distant metastases). The output of the procedure includes a set of *m* selected transcripts and an SVM classifier with associated parameter settings of γ and *C*. A radial basis kernel function exp(−γ ||*x*_i_ - *x*_j_||^2^) is adopted, where *x*_i_ and *x*_j_ are training samples, and γ is a kernel parameter. In this work, γ ∈{2^−7^, 2^−6^, …, 2^8^} and *C*∈{2^−7^, 2^−6^, …, 2^8^}. Each sample is represented using an *n*-dimensional feature vector P = [*p*_1_, *p*_2_, …, *p*_n_]. In this work, *n* = 474. The IGA-chromosome consists of *n* binary IGA-genes *f*_i_ to select features and two 4-bit genes for encoding γ and *C*. The corresponding feature *p*_i_ (the *i*-th transcript) is excluded from the SVM classifier if *f*_i_ = 0, and is included if *f*_*i*_ = 1. Let *m* be the sum of *f*_*i*_. The IBCGA with the fitness function *f*(*G*) that uses LOOCV can simultaneously obtain a set of solutions, *S*_*r*_, where *r* = *r*_start_, *r*_start_ + 1, …, *r*_end_ in a single run. In this work, the parameter settings are *r*_start_ =10, *r*_end_ =30, *N*_pop_ =60, *p*_*c*_ =0.8, *p*_*m*_ =0.05, and *Gmax* =60. The customized IBCGA for transcript selection is given below.

Step 1 (Initiation) Randomly generate an initial population of *N*_pop_ individuals. All *n* binary genes *f*_i_ have *r* 1 s and *n*-*r* 0 s where *r* = *r*_start_.

Step 2 (Evaluation) Evaluate the fitness values of all individuals using *f*(*G*).

Step 3 (Selection) Use a conventional method of tournament selection that selects the winner from two randomly selected individuals to generate a mating pool.

Step 4 (Crossover) Select *p*_*c*_ · *N*_pop_ parents from the mating pool to perform orthogonal array crossover [25] on selected pairs of parents where *p*_*c*_ is the probability of crossover operations.

Step 5 (Mutation) Apply a conventional mutation operator to the randomly selected *p*_*m*_ · *N*_pop_ individuals (except the best individual) in the new population where *p*_*m*_ is the probability of mutation operations.

Step 6 (Termination test) If the stopping condition (reaching *Gmax* generations) for obtaining the solutions *S*_*r*_ is satisfied, then output the best individual as *S*_*r*_. Otherwise, go to Step 2.

Step 7 (Inheritance) If r < *r*_end_, then randomly change one bit in the binary genes *f*_i_ for each individual from 0 to 1; increase *r* by one, and go to Step 2.

Step 8 (Non-deterministic) Perform Steps 1–7 for *R* (=30 in this work) independent runs and obtain the best of the *R* solutions. The best solution can be determined by considering the most accurate one (*S*_*a*_) with the highest fitness value or the robust one (*S*_*b*_) with the highest score of appearance [27]. The appearance score considers both the fitness value as well as the mean number of times for individual genes selected in the *R* runs.

Notably, all genetic algorithms search for globally optimal solutions but their outputs are non-deterministic because of randomization. Therefore, the common approach to solving with the non-deterministic problem is to perform a number of independent runs to evaluate the final answer. In this work, the answers obtained in all *R* runs are utilized efficiently, as described in the next section.

#### Gene selection using a sequential backward selection method

An IGA-based gene selection method with SVM (known as ESVM) [19] can obtain a high prediction accuracy of 96.88% with a mean number of 10.0 to select genes from 11 benchmark datasets concerning various cancers using 10-fold cross-validation (10-CV). For two two-class tumor datasets, ESVM yields a mean number of 4.65 and a classification accuracy of 97.82%. An IGA-based gene selection method uses a maximum likelihood (MLHD) classifier [20] to select a minimal number of relevant genes for accurate classification of tumor samples. The experimental results show that the hybrid method IGA/MLHD outperforms existing methods in terms of the number of selected genes (9.86 on average), classification accuracy (mean accuracy of 96.20%), and robustness of the selected genes based on 11 human cancer-related gene expression datasets.

In this work, the IBCGA that is based on IGA with an SVM selects a small set of genes that are relevant to the distant metastasis of lung cancer while maximizing the fitness function. Since the number of samples (78) is very small, the IBCGA can identify a very small number *m* of genes and obtain a very high training accuracy with *m* < 10. However, a very small number of genes can provide a very high training accuracy (either LOOCV or 10-CV) but the independent accuracy is not always satisfactory owing to overtraining. Moreover, a feasible set of biomarkers for yielding the high tumor prediction accuracy on an independent test dataset often has more than 10 genes. Therefore, this work proposes a novel methodological approach to alleviating the overtraining problem in two ways: 1) by utilizing additional clinical data, i.e. the disease-free survival curve, and 2) using a sequential backward selection (SBS) method to select the best set of transcripts from all of the selected transcripts of the *R* = 30 runs of the IBCGA. For each run, the IBCGA selects at least 10 transcripts (*r*_start_ = 10). Sequential backward selection starts from the full set of the 30 transcripts with the highest appearance times, and sequentially removes transcript x from G (called the G-x set) that results in the smallest decrease of the value of the objective function *f*(*G*-x). Notably, the removal of a feature may actually increase the value of the objective function such that *f*(*G*-x) > *f*(*G*).

## Results and discussion

### Performance evaluation with various weights

To determine the best value of the weight *w* and prevent overtraining in the subsequent design of gene selection methods, all the 78 samples are randomly divided into five groups, of which four are used as a training set and the other serves as an independent test set. Each group of samples serves as a test set in turn. Four experiments with *w* values of 1.0, 0.8, 0.5, and 0.2 are conducted. Table 2 shows the mean accuracies of the independent test for various weights. The results reveal that the best performance is achieved using *w* = 0.8 with a test accuracy of 74.82%. The conventional method has an accuracy of 61.62% when the disease-free survival area is not used (*w* = 1.0). The additional use of a survival curve can compensate for the shortcomings of insufficient microarray samples and improve the test accuracy 13.20%.

**Table 2:** **Accuracies of the independent test for various weights in the fitness function**

**Weight** ***w***	**Test accuracy (%)**
1.0	61.62
0.8	74.82
0.5	62.15
0.2	51.69

### Identifying a gene set using IBCGA and sequential backward selection

**Figure 2:**
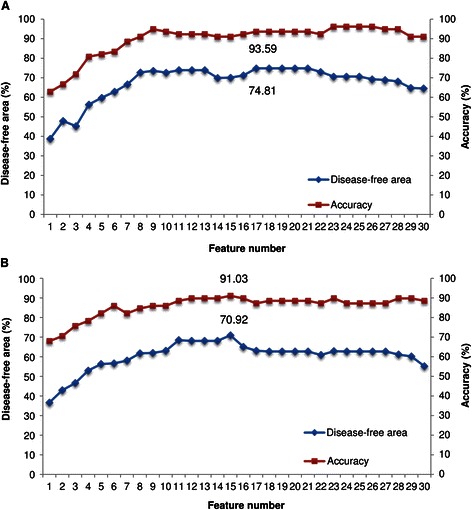
**The prediction accuracy and disease-free survival area obtained using the sequence backward selection method. (A)** *w* = 0.8 **(B)** *w* = 1.0.

The IBCGA identifies a small number *m* of transcripts from *n* = 474 candidate transcripts while maximizing the fitness function *f*(G). The results show that *m* = 10 has a very high value of *f*(G). The IBCGA with *R* = 30 runs yields 30 sets of transcripts. The most accurate solution *S*_*a*_ and the most robust solution *S*_*b*_ are recorded. The number of appearances of each selected transcript in the 30 runs is recorded. Table 3 lists statistical results concerning the number of appearances for the 30 transcripts with the highest appearance frequency. The genes with rank 1 are FBJ murine osteosarcoma viral oncogene homolog B (*FOSB*) and microtubule associated serine/threonine kinase 1 (*MAST1*), which were selected 12 times. The gene forkhead box E1 (*FOXE1*) has two transcripts with ID numbers 6250309 and 3450692, named FOXE1-1 and FOXE1-2, at ranks nine and 13, respectively. Similarly, the gene protein kinase C beta (*PRKCB1*) has two transcripts with ID numbers 3460564 and 5090563, named PRKCB1-1 and PRKCB1-2, at ranks 23 and 27, respectively. The set of 30 transcripts was used in the sequential backward selection (SBS) method for further identifying a set of prognostic biomarkers that are effective and stable in predicting lung cancer metastasis. Figure 2 shows the results of the SBS method for *w* = 0.8 and 1.0. Table 4 shows the performance of the IBCGA and SBS methods for *w* = 0.8 and 1.0.

**Table 3:** **The 30 top-ranked transcripts in terms of selected times from 30 runs**

**Rank**	**Transcript**	**Frequency**	**Rank**	**Transcript**	**Frequency**
1	FOSB	12	16	CDKL5	3
2	MAST1	12	17	DCK	3
3	CCL15	8	18	KLF12	3
4	MAK	6	19	ZAP70	3
5	SF1	5	20	BACH2	3
6	HDAC9	5	21	YSK4	3
7	YSK4	4	22	ELF5	3
8	EDN1	4	23	PRKCB1-1	2
9	FOXE1-1	4	24	CEP110	2
10	GLI3	4	25	HS.541237	2
11	CDKN2AIP	4	26	KLF6	2
12	CREG1	3	27	PRKCB1-2	2
13	FOXE1-2	3	28	MAK	2
14	CSNK1A1	3	29	CCL16	2
15	TUB	3	30	IL23A	2

**Table 4:** **The performance of the IBCGA and sequential backward selection (SBS) methods**

**Method**	***w*** **= 0.8**	***w*** **= 0.8**	***w*** **= 1.0**	***w*** **= 1.0**
	***Acc*** **(%)**	***Asurv*** **(%)**	***Acc*** **(%)**	***Asurv*** **(%)**
IBCGA (*S*_*a*_)	95.95	70.01	97.43	69.21
IBCGA (*S*_*b*_)	92.02	63.81	94.73	66.91
SBS	93.59	74.81	91.03	70.92

From Figure 2, the SBS method with 17 transcripts and *w* = 0.8 has the largest disease-free survival area (*Asurv* = 74.81%) and prediction accuracy (*Acc* = 93.59%). The largest *Asurv* is 70.92% and *Acc* = 91.03% when a set of 15 transcripts was adopted for *w* = 1.0. From Table 4, the *S*_*a*_ solution has a set of 10 transcripts and is associated with a performance of *Acc* = 95.95% and *Asurv* = 70.01% for *w* = 0.8. The *S*_*b*_ solution has a set of 10 transcripts and is associated with a performance of *Acc* = 92.02% and *Asurv* = 63.81% for *w* = 0.8. Consider that 1) the 30 transcripts with high frequency are selected; 2) the test accuracy of *w* = 0.8 is higher than that of *w* = 1.0; and 3) the set of 17 transcripts yields the largest disease-free survival area. Therefore, Table 5 presents the 17 transcripts of the 16 genes obtained using the SBS method with *w* = 0.8. The 16 genes regarded as a candidate set of biomarkers (the 16-gene set) can be further analyzed.

**Table 5:** **The 17 transcripts obtained by the sequence backward selection method with the disease-free survival area**

**Transcript**	**Classification ability% (Rank)**	**Disease-free survival area% (Rank)**	**Rank (p-value)**
CCL16	74.36 (1)	43.58 (2)	5
GLI3	71.79 (2)	34.43 (6)	15
TUB	71.79 (3)	35.71 (4)	13
PRKCB1-1	70.51 (4)	34.51 (5)	3
ZAP70	67.95 (5)	29.53 (8)	14
ELF5	67.95 (6)	26.70 (10)	17
EDN1	66.67 (7)	31.09 (7)	9
SF1	66.67 (8)	25.87 (11)	8
CREG1	66.67 (9)	23.40 (14)	2
MAST1	65.38 (10)	23.71 (13)	12
CSNK1A1	64.10 (11)	24.95 (12)	11
HDAC9	64.10 (12)	27.78 (9)	10
MAK	62.82 (13)	38.76 (3)	4
CCL15	61.54 (14)	22.94 (15)	6
PRKCB1-2	61.54 (15)	16.73 (17)	1
CDKN2AIP	61.54 (16)	46.00 (1)	16
FOXE1-1	60.26 (17)	19.60 (16)	7

### Analyzing identified 16-gene set

To investigate the abilities of individual genes to predict distant metastasis, the rankings by classification ability, disease-free survival area, and p-value using the training dataset are analyzed. The classification ability ranking is derived according to the classification accuracy using a single gene and the SVM classifier with a parameter setting using a grid search method. Table 5 lists the rankings of classification ability, disease-free survival area, and p-value for every transcript in the 16-gene set. Table 5 reveals that the distributions of rankings for the three metrics are diverse. All of the p-values of the 17 transcripts are very small so a comparison of p-value ranking is not meaningful. The Person correlation coefficient between the ranks based on the two metrics (accuracy and survival area) is not very high (*R* = 0.4755). The gene chemokine (C-C motif) ligand 16 (*CCL16*) provides the highest classification accuracy (74.36%) and the second largest area of disease-free survival (43.58%). The gene CDKN2A-interacting protein (*CDKN2AIP*) has a disease-free survival area of 46.00% (rank 1) and a classification accuracy of 61.54% (rank 16). The significant variation among the rankings of genes based on the three metrics is discussed below.

The effective discrimination between distant and non-distant metastases can be achieved using a set of interacted genes that are involved in various signaling pathways, rather than a set of mutually independent genes. The LOOCV accuracy of 93.59% that is obtained using the 16-gene set substantially exceeds that obtained using a single gene. On the other hand, when all of the 17 transcripts are used to predict distant metastasis, the disease-free survival area (*Asurv*) is 74.81%, which is very close to the real area of 73.21%. Moreover, this area (74.81%) is much larger than that obtained using a single gene. The expression level of individual genes using the microarray technique cannot be used reliably to discriminate between samples of distant and non-distant metastases. Several factors determine patient survival, including gene expression and the nature of the therapy. Therefore, survival curve provides valuable information, but care must be taken in using the survival curves of individual genes. In brief, the rankings of individual genes for the three metrics based on univariate analysis can be used in initial screening in coarse-to-fine gene selection. In this work, the discovery of biomarkers in the fine stage takes into account a set of genes by combining both the classification ability and disease-free survival area.

Numerous prediction methods discover biomarkers by searching for a set of genes that can provide highly accurate performance of LOOCV or 10-CV [19,20]. Many sets may comprise a small number of genes that can achieve the same goal. The highest LOOCV accuracy that can be achieved using SVM with ten genes without considering the disease-free survival curves is 97.43% (*w* = 1.0, Table 4). The proposed method of applying SBS to a combination of promising gene sets aims to identify a set of reliable biomarkers for predicting distant metastasis. Notably, some genes in the set of 30 transcripts (Table 3) but not in the identified 16-gene set may also be potential biomarkers.

### Evaluation of biomarkers using an independent cohort

To evaluate generalizability of the identified gene sets, an independent cohort of 26 samples was utilized. Table 6 shows the performance of the IBCGA and the SBS methods by performing 30 runs on the 26 test samples. The training accuracies for *w* = 1.0 and 0.8 are 88.80% and 88.88%, respectively, which are very close to each other. However, the test accuracies for *w* = 1.0 and 0.8 are 50.41% and 61.08%, respectively. The test accuracy obtained using disease-free survival curves (*w* = 0.8) is larger than that obtained using no survival curve (*w* = 1.0) that is improved by 10.67%. The test accuracies of the SBS method for *w* = 1.0 and 0.8 are 53.84% and 65.38%, respectively. The improvement in the test accuracy is 11.54%. The improvement in the mean test accuracy using the disease-free survival curves is 11.10%. The results also reveal that the SBS method that includes the IBCGA outperforms the IBCGA method alone for both *w* = 1.0 and 0.8.

**Table 6:** **The test accuracies of the IBCGA and sequence backward selection (SBS) methods from 30 runs**

**Method**	**No. of features**	**Training** ***Acc*** **(%)**	**Test** ***Acc*** **(%)**
IBCGA (*w* = 1.0)	10	88.80 ± 3.92	50.41 ± 6.56
IBCGA (*w* = 0.8)	10	88.88 ± 3.27	61.08 ± 7.35
SBS (*w* = 1.0)	15	91.03	53.84 (14/26)
SBS (*w* = 0.8)	17	93.59	65.38 (17/26)
SVM ensemble	17	93.59	76.92 (20/26)

The SBS method with *w* = 0.8 is the best method, yielding an LOOCV accuracy of 93.59% and a predicted disease-free survival area of 74.81%, which is very close to the real disease-free survival area of 73.21%. The accuracy of the independent test using an SVM classifier with 17 transcripts is 65.38%. The SVM ensemble of 30 SVM classifiers of which each uses five transcripts that are randomly selected from the 17 transcripts can yield the test accuracy of 76.92%. The intractable problem of overtraining has been alleviated by the additional use of survival curves in the proposed optimization method. However, this performance should be not sufficient to select a putative set of biomarkers. Increase of the number of samples can further mitigate the overtraining problem.

### Performance comparison of various gene sets

Table 7 shows the type of regulator and location of its protein product, as well as the related cancer genes in the 16-gene set. The 16 genes are as follows: 1) endothelin 1 (*EDN1*), 2) casein kinase 1, alpha 1 (*CSNK1A1*), 3) chemokine (C-C motif) ligand 15 (*CCL15*), 4) splicing factor 1 (*SF1*), 5) gene protein kinase C beta (*PRKCB1*), 6) microtubule-associated serine/threonine protein kinase 1 (*MAST1*), 7) zeta-chain associated protein kinase 70 kDa (*ZAP70*), 8) chemokine (C-C motif) ligand 16 (*CCL16*), 9) E74-like factor 5 (*ELF5*), 10) CDKN2A-interacting protein (*CDKN2AIP*), 11) histone deacetylase 9 (*HDAC9*), 12) GLI family zinc finger 3 (*GLI3*), 13) forkhead box E1 (*FOXE1*), 14) male germ cell-associated kinase (*MAK*), 15) tubby protein homolog (*TUB*), and 16) cellular repressor of E1A-stimulated genes 1 (*CREG1*). The 16-gene set can be categorized into two subsets. One subset has five known lung-cancer-related genes (named 5-gene set), including *EDN1*, *CSNK1A1*, *CCL15*, *SF1* and *PRKCB1*. The other subset (11-gene set) consists of nine cancer-related genes and two potential biomarkers (*TUB* and *CREG1*).

**Table 7:** **The type of regulator and location of its protein product, as well as the related cancer of the genes in the 16-gene set**

**No**	**Gene name**	**Type of regulator**	**Location**	**Related cancer**
1	*EDN1*	Cytokine	Extracellular space	Lung cancer breast cancer
2	*CSNK1A1*	Kinase	Cytoplasm	NSCLC
3	*CCL15*	Cytokine	Extracellular space	NSCLC
4	*SF1*	Transcription regulator	Nucleus	Lung cancer
5	*PRKCB1*	Kinase	Cytoplasm	Lung cancer
6	*MAST1*	Kinase	Cytoplasm	Breast cancer
7	*ZAP70*	Kinase	Plasma membrane	Colorectal cancer
8	*CCL16*	Cytokine	Extracellular space	Mammary Adenocarcinoma
9	*ELF5*	Transcription regulator	Nucleus	Cancer
10	*CDKN2AIP*	Transcription regulator	Nucleus	Cancer
11	*HDAC9*	Transcription regulator	Nucleus	Medulloblastoma
12	*GLI3*	Transcription regulator	Nucleus	Cancer
13	*FOXE1*	Transcription regulator	Nucleus	Thyroid
14	*MAK*	Kinase	Cytoplasm	Prostate cancer
15	*TUB*	Transcription regulator	Cytoplasm	Relation to ocular diseases
16	*CREG1*	Transcription regulator	Nucleus	Inhibitor of apoptosis

A number of lung cancer-related genes in the literature are considered for further comparison and analysis. Since EMT is known to be involved in tumor malignancy, some mesenchymal-related genes of gastric cancer, including *WNT5A*, *CDH2*, *PDGFRB*, *EDNRA*, *ROBO1*, *ROR2*, and *MEF2C* that are activated by an EMT regulator, are also examined [28]. Table 8 presents the 32 genes and their relevant papers [28-39]. The same sequence backward selection (SBS) method is applied to the 32-gene set, yielding a set of nine genes that can be used to accurately predict distant metastasis of lung cancer. The 9-gene set consists of *MEF2C*, *MMP-2*, *ID2*, *CDH2*, *WNT5A*, *CDH1*, *TGFB1*, *MMP-9*, and *TWIST2*. Notably, cigarette smoke induces *WNT5A*-coupled PKC activity during lung carcinogenesis, which causes Akt activity and anti-apoptosis in lung cancer [40]. The two mesenchymal-related genes, *CDH2* and *MEF2C* were also selected into the 9-gene set.

**Table 8:** **The 32 lung cancer-related genes and their relevant papers**

**No.**	**Gene name**	**Paper**
1	*CDH2, PDGFRB, ROBO1, ROR2, MEF2C, WNT5A, EDNRA*	Ohta *et al.* [28]
2	*ZEB1, ZEB2, CDS1, ST14, FGFR1, TWIST1, TWIST2, TGFB1*	Gemmill *et al*. [29]
3	*VIM, MUC1, S100A4, FOXQ1*	Feng *et al.* [30]
4	*ID2, TGFB1*	Yoshikawa *et al.* [31]
5	*SNAI1, MMP-9, MMP-7, MMP-2, S100A4, TGFB1*	Ward *et al.* [32]
6	*HMGA2, TTF1*	Qi *et al.* [33]
7	*SYK*	Singh *et al.* [34]
8	*TYRO3, AXL, PDGFRB*	Thomson *et al.* [35]
9	*SIP1, ZEB1*	Takeyama *et al.* [36]
10	*TCF4*	Xiang *et al.* [37]
11	*SNAI1, TGFB1*	Matsuno *et al.* [38]
12	*CDH1, TWIST1*	Pallier *et al.* [39]

Table 9 presents performance comparisons of various gene sets in terms of the prediction accuracy. The training accuracy, disease-free survival area, and independent test accuracy obtained using the 32-gene set are 65.38%, 34.47%, and 50.00%, respectively. The performance of the 32-gene set is not good, especially for the independent test. This lung cancer-related gene set is not designed especially to predict distant metastasis of lung cancer. The 9-gene set yields a training accuracy of 73.07%, a disease-free survival area of 55.70%, and an independent test accuracy of 80.76%. As a result, the 9-gene set that is obtained using the SBS method is more effective in identifying the distant metastasis than is the 32-gene set, and is worthy of further validation.

**Table 9:** **Performance comparison among various gene sets**

**Gene set**	**Training** ***Acc*** **(%)**	**Training** ***Asurv*** **(%)**	**Test** ***Acc*** **(%)**
32-gene (lung cancer related)	65.38	34.47	50.00 (13/26)
9-gene (SBS from the 32-gene set)	73.07	55.70	80.76 (21/26)
16-gene (EMT related, SVM ensemble)	93.59	74.81	76.92 (20/26)
11-gene (EMT and cancer related)	87.18	53.37	69.23 (18/26)
5-gene (EMT and lung cancer related)	78.25	52.33	76.92 (20/26)
1-gene (*CCL16*, maximum *Acc*)	74.36	43.58	57.69 (15/26)
1-gene (*CDKN2AIP*, maximum *Asurv*)	61.54	46.00	76.92 (20/26)

The value of *Asurv* is 73.21% for real classes of samples in the training dataset.

**Figure 3:**
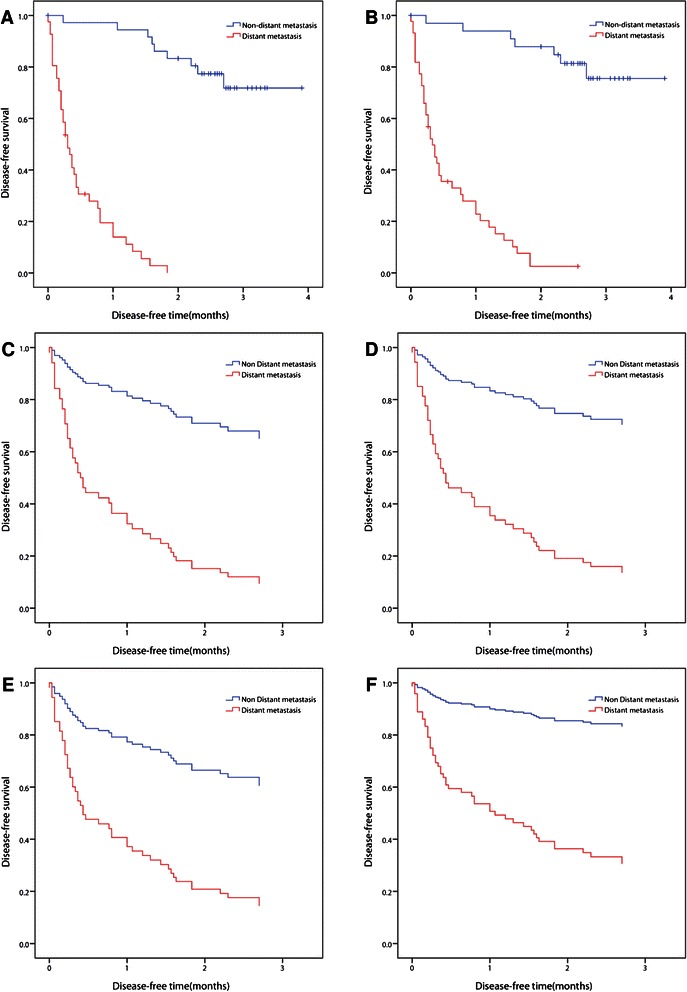
**The disease-free survival areas of various gene sets using Kaplan-Meier survival curves.** There are 37 non-distant- (in blue) and 41 distant-metastasis samples (in red). **(A)** real class (73.21%), **(B)** 16-gene set (74.81%), **(C)** 11-gene set (53.37%), **(D)** 5-gene set (52.33%), **(E)** *CCL16* (43.58%) and **(F)** *CDKN2AIP* (46.00%).

The 16-gene set for predicting distant metastasis is identified using microarray and survival data at the same time. The test accuracy is 76.92% for the 16-gene set using an SVM ensemble classifier. To analyze the 16-gene set further, its two subsets (5- and 11-gene sets) are independently evaluated using the same prediction method as that used to evaluate the 16-gene set, and the results are shown in Table 9. The training accuracy, disease-free survival area, and independent test accuracy achieved using the 5-gene set with six transcripts are 78.25%, 52.33%, and 76.92%, respectively, which are close to those achieved using the 9-gene set. The 11-gene set of EMT and cancer-related genes has a higher training accuracy of 87.18%, which is nearly equal to the survival area of 53.37%, and has a smaller test accuracy of 69.23% than the 5-gene set. To compare individual genes in terms of their prediction performance, three representative genes in the 16-gene set were selected. The top genes with the highest training accuracy and disease-free survival area are *CCL16* (74.36%) and *CDKN2AIP* (46.00%), respectively. Figure 3 plots the Kaplan-Meier survival curves and their corresponding disease-free survival areas for real and predicted classes obtained using various gene sets.

**Figure 4:**
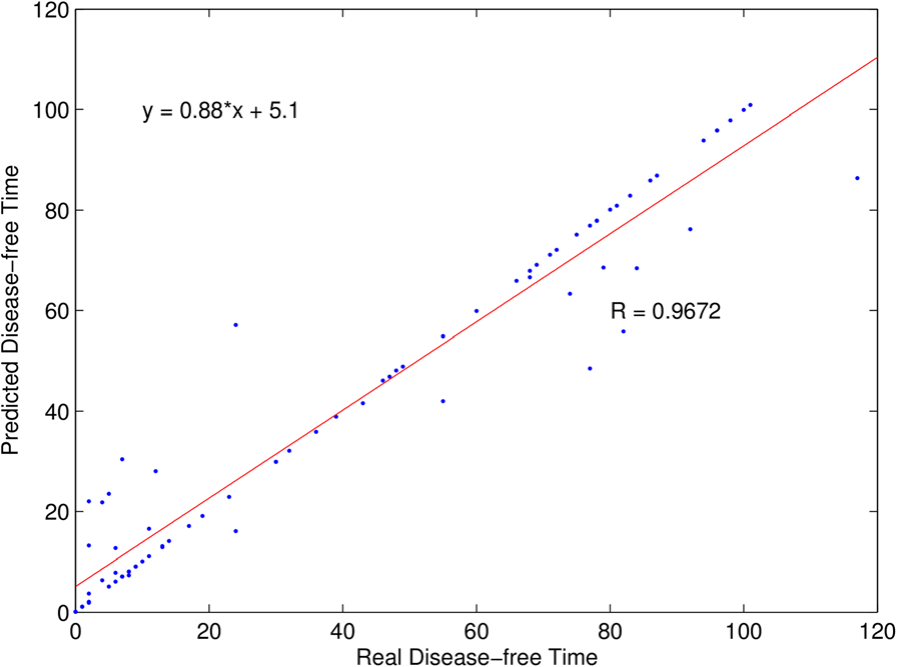
**The predicted disease-free survival time using the support vector regression with the 16-gene set.**

The large disease-free survival area (74.81%) of predicted classes, obtained using the 16-gene set, is remarkably very close to that of the real classes (73.21%). To investigate the ability of this set to predict patient’s disease-free survival time, the support vector regression in the LIBSVM [41] is used to establish a survival prediction model. Figure 4 shows that, in the estimate of the disease-free survival time, the correlation coefficient between the real and predicted disease-free survival times is *R* = 0.9672. The mean survival prediction error is 3.99 months. This result reveals that the 16-gene set is also effective in predicting disease-free survival times of patients.

### Examination of the 16 putative biomarkers

The 16 putative biomarkers herein are examined with reference to the relevant papers. Five of the 16 genes have been shown to be related to lung cancer progression. Decreased expression of *EDN1* has been reported in primary lung cancers, possibly owing to the high methylation in the CpG island of its intron 1 and exon 2 junction [42]. Overexpressions of *EDN1* and *EDNRA* were already reported as being associated with impaired survival in human breast cancer [43]. Expression analysis reveals that hypoxia-induced lung cancer related-biomarkers *HIF* and its modulating proteins, including *CSNK1A1*, are significantly down-regulated [44]. The *CCL15* level correlates with response to combination therapy with erlotinib and celecoxib in patients with NSCLC [45]. Chemokine *CCL15* is the most significant marker that is associated with increased odds of short survival [46]. Splicing factor *SF1* participates in the ATP-dependent formation of the spliceosome complex [47]. Down-regulation of the oncogenic serine/arginine-rich splicing factor 1 (*SF1*) leads to the skipping of an exon that is overexpressed in primary lung tumors [48]. *PRKCB* belongs to a family of serine/threonine-specific kinases and is predominantly activated by diacylglycerol, calcium, and phorbol ester. The two splice-variants are called *PRKCB1* and *PRKCB2. PRKCB1* exists in lung cancer cell lines in the context of enzastaurin-induced proliferation and kinase inhibition, determined by using exon sequencing, immunoblotting, and cytotoxicity assays in NSCLC and SCLC cell lines [49].

The nine cancer-related genes are briefly described below. Breast cancer cell lines that harbor Notch gene rearrangements are uniquely sensitive to the inhibition of Notch signaling, and the overexpression of *MAST1* or *MAST2* gene fusions has a proliferative effect [50]. The overexpressed gene *ZAP70* significantly relates to prognostic factors such as tumor size, advanced stage, invasive depth, lymph node metastasis and differentiation [51]. An adenovirus that encodes *CCL16*, when injected into established nodules significantly delayed tumor growth [52]. *ELF5* can transactivate through the sequences of ETS transcription factors. *ELF5* is localized to human chromosome 11p13-15, which is a region that frequently undergoes loss of heterozygosity in several types of carcinoma, including those of the breast, kidney and prostate [53]. *CDKN2AIP* has a known role in tumorigenesis [54]. A functional role of *HDAC5* and *HDAC9* in tumor cell growth in medulloblastoma cell lines has been reported [55]. Three sonic hedgehog effectors, *GLI1*, *GLI2*, and *GLI3*, regulate the transcription of diverse genes that are involved in cell growth and cell proliferation [56]. *FOXE1* (TTF-2) is a thyroid-specific transcription factor and a marker in thyroid tumors. The lung is the most common distant metastatic site for thyroid carcinomas [57]. *MAK* is a direct transcriptional target of androgen receptor which plays an important role in the normal development of prostate as well as in the progression of prostate cancer [58]. Phylogenetically, *TULP3* is the family member that is most closely related to *TUB. TULP3* is detected at high levels in human RNA from testes, ovaries, the thyroid, and the spinal chord [59]. *TUB* encodes a protein of 561 amino acids that is highly expressed in a number of tissues examined, including the heart, brain, ovary, thyroid, spinal chord, and retina and it maps to chromosome 11p15.4 [60]. The overexpression of *CREG1* reduces cell proliferation in immortal LFS and cancer cell lines. *CREG1* has been identified as a potent inhibitor of apoptosis [61]. The cooperation of *CREG1* and p16 (INK4a) inhibits the expression of cyclin A and cyclin B by inhibiting promoter activity, reducing mRNA and protein levels, and these proteins are required for S-phase entry and G2/M transition [61,62].

After these putative biomarkers are mapped into the KEGG pathway database (http://www.genome.jp/kegg/), several signaling pathways that involve lung cancer metastasis are identified. Mitogen-activated protein kinase signaling pathway, NF-kappa B signaling pathway, and immune response IL-1 signaling pathway have been reported in lung cancer metastasis. While regulating proliferation, gene expression, differentiation, mitosis, cell survival, and apoptosis, the mitogen-activated protein kinase signaling pathway has long been viewed as an attractive pathway for anticancer therapies [63]. A higher nuclear factor-kappa B expression pattern is associated with more advanced stages of oncogenesis and it will expand related pathways for invasion and metastasis in lung cancer [64]. Abnormal IL-1 expression and its related pathway seem to be related to IKK alpha/beta activation, p65 translocation and transcription activity, and associated to migration of cancer cells [65]. We suggest that the pathways that are associated with these biomarkers might play an important role in NSCLC metastasis. This inference offers an opportunity to expand greatly our knowledge of the expression patterns in NSCLC. Considering together the expression status of biomarkers, related signaling pathways, and clinical outcome will further reveal the roles of the biomarkers in lung cancer metastasis. An enhanced understanding of the basic biological mechanisms of NSCLC will likely facilitate development of improved methods for survival prediction.

## Conclusions

This work proposes a novel methodological approach to discovering a set of prognostic biomarkers for predicting the distant metastasis of lung cancer through simultaneous utilization of microarray and survival data. The presented optimization method uses an objective function to maximize both prediction accuracy and the disease-free survival area to identify a set of biomarkers. The additional use of clinical disease-free survival time can greatly facilitate the discovery of biomarkers and the prediction of survival time. The proposed method that combines both microarray and survival data can also alleviate the problem of overtraining that arises from the insufficiency of samples. The experimental results herein show that a combination of multiple biomarkers may increase diagnostic sensitivity and specificity over those obtained using individual biomarkers. The proposed method has high generalizability in the discovery of prognostic biomarkers not only for the distant metastasis of lung cancer, but also for other cancers using microarray data and clinical outcomes.

Few studies have investigated the discovery of prognostic biomarkers for predicting the distant metastasis of lung cancer. This work identified a set of 16 prognostic biomarkers that can be used to predict distant metastasis with high accuracies, a leave-one-out cross-validation accuracy of 93.59%, an independent test accuracy of 76.92%, a large predicted disease-free survival area of 74.81% (close to 73.21% for real survival area), and a mean survival prediction error of 3.99 months. Closely examining the 16-gene set by mapping these genes into the KEGG pathway database reveals some signaling pathways that are involved in lung cancer metastasis. Future work will incorporate these findings of signaling pathways with related biomarkers and clinical outcomes to develop novel methods for predicting distant metastasis and the survival times of patients with early-stage NSCLC.

## Additional files

Additional file 1: Table S1. The data set of 78 lung cancer patients for training.
Additional file 2: Table S2. The data set of 26 lung cancer patients for test.
